# Evaluation of short training session for venous limited compression ultrasonography: prospective multicenter study

**DOI:** 10.1186/s13089-020-0155-2

**Published:** 2020-02-03

**Authors:** François Javaudin, Julie Seon, Quentin Le Bastard, Astrid Cabiot, Philippe Pes, Idriss Arnaudet, Milena Allain, Winfocus-France Study Group, Philippe Le Conte

**Affiliations:** 1 Emergency Department, University Hospital, Nantes, France; 2 Emergency Department, Departmental Hospital, La Roche Sur Yon, 44035 Nantes Cedex 01, France

**Keywords:** Emergency, Ultrasound, Ultrasound in medical education, Vascular ultrasound

## Abstract

**Background:**

Venous limited compression ultrasonography (VLCU) is recommended in case of suspicion of deep venous thrombosis (DVT). Current training pathways are rather long and include experiential phase. This aim of this study was to investigate the efficacy of a short training session for VLCU without experiential phase. The training session was applied in residents without previous ultrasound skills. Program included operation of ultrasound device and interpretation of venous images. Included patients were older than 18 years and had a suspicion of DVT. After realization of VLCU using usual technique, residents reported the dynamic compressibility of the femoral and popliteal veins, the presence or not of a visible thrombus, self-reported difficulty and duration. Patients then underwent a whole leg ultrasonography (WLCU) in the local laboratory which was blinded to VLCU results. The main criterion was the negative-predictive value (NPV) of VLCU for the absence of proximal DVT diagnosed with WLCU. Secondary criteria were VLCU diagnostic performances, rate of inability to conclude, difficulty and duration. For a NPV of 95 ± 6%, the needed number of patients was 96. This study was approved by the ethical committee.

**Results:**

102 patients were analyzed. 46 residents were trained. A DVT was diagnosed by WLCU in 18 patients (prevalence of 17.6% [95% CI 11–26%]). VLCU detected 15 DVT (NPV of 96% [95% CI 89–99%]). The positive likelihood ratio was 9.9, the negative likelihood ratio 0.19 and Cohen’s Kappa 0.62 [95% CI 0.52–0.71]. The sensitivity was 83% [CI 95% 60–94%] and specificity 88% [CI 95% 79–93%]. The mean number of VLCU by residents was 2.3 ± 2.1, median 2 (minimum 1, maximum 8). Mean duration was 3.4 min, difficulty was 3.7 ± 2.

**Conclusion:**

The principal objective, NPV 96% [95% CI 89–99%], was achieved. However, this short training session was inadequate to allow ruling-out a DVT with sufficient security. Thus, the experiential phase seems to be essential.

## Background

Venous limited compression ultrasonography (VLCU) is recommended by the American College of Emergency Physicians [1] and by other Societies like the French Society of Emergency Medicine [2] in the evaluation of suspicion of deep venous thrombosis (DVT). This simplified exam coupled with d-dimer testing was successfully evaluated versus whole leg color-coded Doppler ultrasonography (WLCU) in a randomized controlled trial [3]. Actually, isolated distal DVT, below popliteal veins, have a very low rate of extension or pulmonary embolism [4] even in absence of anticoagulant treatment. WLCU needs experienced operators and is most frequently impossible after hours. These reasons have promoted usage of VLCU in the Emergency Departments (ED) since (I) patients with suspected DVT can be admitted 24 h/7 days a week and (ii), this technique can be safely, quickly and accurately performed by Emergency Physicians (EP).

However, the initial training in Point-of-Care Ultrasound (POCUS) of EP remains a crucial question. The American College of Emergency Physicians has formalized the training pathway for EP without previous ultrasound skills [1]. It begins with a didactic course followed by an experiential phase of supervised ultrasounds. A similar POCUS training pathway is proposed in United Kingdom [5]. These training pathways are rather long and time-consuming. A recent Spanish study demonstrated that a 10-h training session provided good results in term of diagnosis accuracy in particular for VLCU [6].

Our goal was thus to investigate a shorter training session, 2-h, without experiential phase for VLCU by performing a prospective study.

## Patients and methods

### Overall

We performed a prospective multicenter observational study to assess the efficacy of this training session for residents without previous ultrasound skills. The main criterion was the negative-predictive value of VLCU since the major objective of this exam in Emergency Medicine is to safely discharge patients without anticoagulants.

### Patients

Inclusion criterion was a suspicion of DVT in patients older than 18 years in the three ED involved in this study. Exclusion criteria were a past history of DVT, symptoms for more than 4 weeks and documented end-of-life precluding investigations.

### Methods

#### Participating physicians and training session

Participating first or second year residents were recruited in three ED. Inclusion criteria were the absence of previous POCUS exposure before participation to our study, in particular, no POCUS course during their medical school nor during their Emergency Medicine residency. They committed themselves not to follow another POCUS training until conclusion of the study. The training session was a standardized 2-h meeting including up to 7 residents with a registered POCUS trainer. The program included operation of ultrasound device, venous compression theory and technique, interpretation of normal and pathological venous images. Approximately half of the time was spent performing venous imaging on other participants under supervision of the POCUS trainer. There were eight sessions performed by the same team, residents stayed on average 6 months in the ED. This study was approved by the Ethics Committee of Nantes University Hospital (reference RC15_047).

#### Study flow

After verification of inclusion and exclusion criteria, obtaining oral informed consent and Wells score calculation [7], a VLCU was performed by resident. It was done using a Philips CX50 with a linear probe following the usual technique described in Lee article (2-point technique) [8]. Residents reported the dynamic compressibility of the right and left common femoral and popliteal veins, the presence or not of a visible thrombus, VLCU difficulty and duration. Patients then underwent a WLCU in the local vascular laboratory which was blinded to the VLCU result.

#### Objectives and criteria

The main objective was the ability to exclude a DVT in case of negative VLCU, Secondary objectives were diagnostic performances of VLCU, inability to conclude on compressibility, concordance between the two exams, duration and difficulty.

The main criterion was the full compressibility of the four sites allowing to calculate the negative-predictive value (NPV) of VLCU for the absence of proximal DVT diagnosed with WLCU. For this purpose, when residents were unsure of the compressibility, it was secondarily coded as absence of compressibility. Secondary objectives were sensitivity, specificity, positive-predictive value (PPV), positive and negative likelihood ratio of VLCU, rate of inability to conclude (common femoral and popliteal), Cohen Kappa between VLCU and WLCU, number of VLCU performed by each residents, self-reported duration and difficulty score of VLCU (coded from 1 very easy to 10 impossible).

#### Statistics

For a negative-predictive value of 95% with a confidence interval of 6%, the needed number of patients was 96. For security purpose, we planned to enroll 106 patients.

Data stored in a Microsoft Access^®^ database were analyzed using PASW Statistics^®^. Numerical data were presented as mean and standard deviation, categorical data as percentage with 95% confidence interval. Numerical data were compared using analysis of variance and Student’s *t* test or non-parametric if needed, categorical data by *χ*^2^ test. A *p* value < 0.05 was considered significant.

## Results

**Fig. 1:**
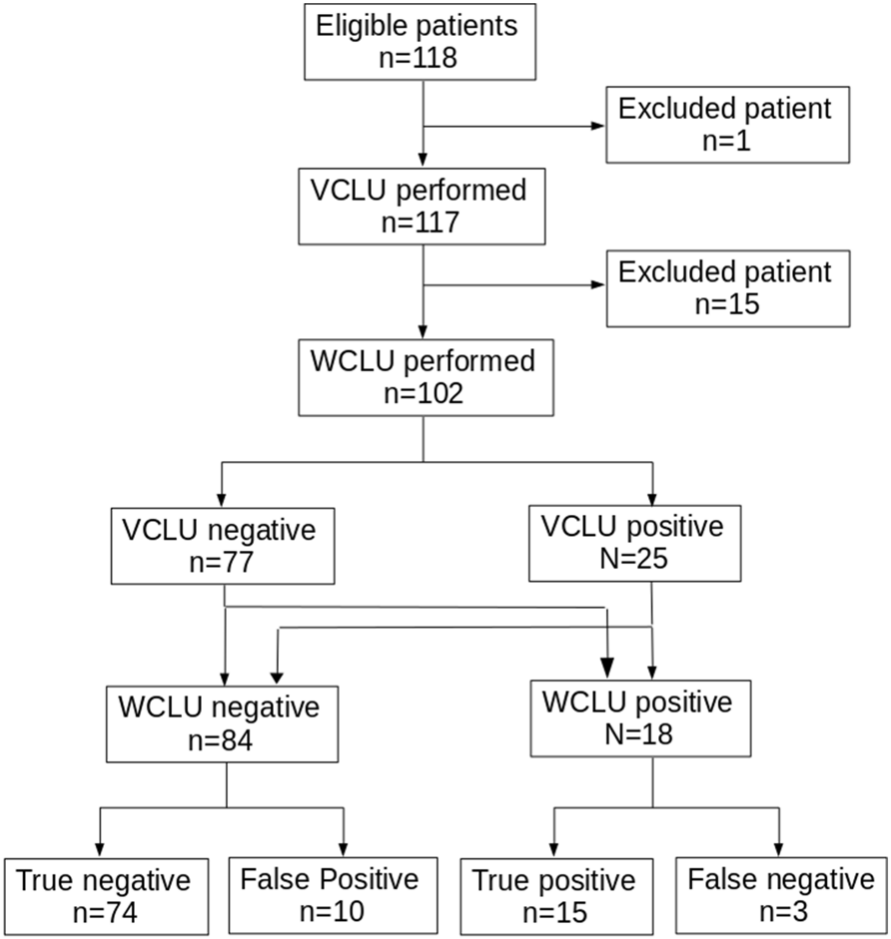
Flowchart of the 102 patients included in the study. *VLCU* venous limited compression ultrasonography, *WLCU* whole leg color-coded Doppler ultrasonography

Between December 2014 and August 2017, 118 patients were included, 16 secondarily excluded (one with a past history of DVT, 15 without WLCU), thus 102 patients were analyzed (Center 1: 50 patients, center 2: 44 and center 3: 8) (Table 1). The flowchart of patients is displayed in Fig. 1.

**Table 1:** Main characteristics and Wells score of the 102 included patients

Variable	Value
Age	59 ± 20 years
Sex	47 women, 55 men
Wells score 0	18 (18%)
Wells score 1–2	46 (45%)
Wells score > 2	34 (33%)
Wells score not done	4 (4%)

During the same time, 46 residents were trained. A DVT was diagnosed by WLCU in 18 patients, leading to prevalence of 17.6% [95% CI 11–26%]. VLCU detected 15 DVT, 3 were missed (two popliteal veins and one femoral) leading to a NPV of 96% [95% CI 89–99%] (contingency table displayed in Table 2).

**Table 2:** Contingency table of diagnostic performances of venous limited compression ultrasonography in 102 patients suspected to have a DVT

	DVT+	DVT−	Total	Predictive values
VCLU+	15	10	25	PPV 60% [CI 95% 41–76%]
VCLU−	3	74	77	NPV 96% [CI 95% 89–99%]
Total	18	84	102	
	Sensitivity 83% [CI 95% 60–94%]	Specificity 88% [CI 95% 79–93%]		

*χ*^2^ 40.8, *p* = 10^−9^

*VLCU* venous limited compression ultrasonography, *DVT* deep venous thrombosis, *PPV* positive-predictive value, *NPV* negative-predictive value

**Fig. 2:**
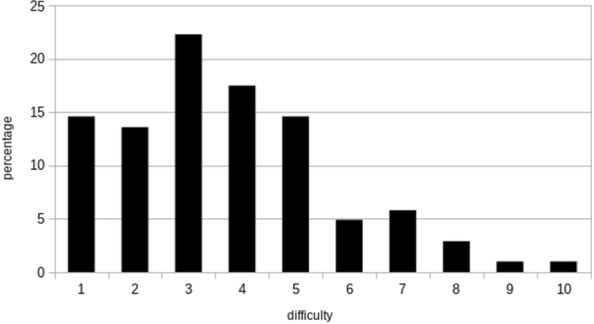
Self-assessed difficulty of venous limited compression ultrasonography performed by 46 residents on 102 patients

The positive likelihood ratio was 9.9, the negative likelihood ratio 0.19 and Cohen’ Kappa 0.62 [95% CI 0.52–0.71]. The sensitivity was 83% [CI 95% 60–94%] and specificity 88% [CI 95% 79–93%]. Residents were unable to conclude on complete compressibility in 13 femoral and 22 popliteal veins χ_2_ test between the two sites, *p* = 0.15), leading to a whole uncertainty *score* of 7.5%. The mean number of VLCU by residents was 2.3 ± 2.1, median 2, (minimum 1, maximum 8). Duration of VLCU was 3.4 ± 2.1 min, difficulty score was assessed as 3.7 ± 2 (Fig. 2).

## Discussion

The principal objective, NPV 96% [95% CI 89–99%], was achieved. However, this short training session was inadequate to allow ruling-out a DVT with sufficient security. Actually, 3 DVT out of 18 were not identified by the residents. A recent guideline of the American Society of Hematology recommends a NPV rate of 2% [9]. Our study was thus not powerful enough. Furthermore, there were also 10 false-positive exams that would have possibly led to an unwarranted anticoagulant treatment. DVT prevalence, 17.6% [95% CI 11–26%], was not different when compared to other studies as reported in a meta-analysis including 2379 patients [10] (23%). Our results showed that performances tend to be less accurate than in a majority of published studies. In the same meta-analysis [10], pooled sensitivity and specificity were 94.8% and 96.2%, respectively, when compared to our results, 83% and 88%, respectively. Nevertheless, there was an overlap between the confidence interval for sensitivity precluding definite answer. Comparison with the Jang [11] study exhibits similar results. However, the goal of this study was to evaluate a very short training session in residents without previous ultrasound skills. In the literature, training was either longer [6, 12, 13] or addressed physicians with previous POCUS experience [11, 14–17]. To the best of our knowledge, it is the first study which assesses performances of a very short training session in venous ultrasound for residents without previous POCUS skills. It is likely that the absence of an experiential phase on real patients was crucial to explain these results. During the training session, residents had only performed normal VLCU on one or two other participants. Confronted with real patients, they assessed a difficulty score at 3.7 ± 2 which was relatively high and were unable to conclude in 7.5% of examined sites. Furthermore, the number of exams per resident was low, 2.3 ± 2.1 which prevented acquisition of diagnostic capacities. This fact could be partially explained by the residents’ duration of stay in the ED.

Our intention is thus to modify our training pathway by including an experiential phase of 15 monitored VLCU by resident. Actually, in an article on learning curves in POCUS, conversely to other sites such as soft tissues or Focused Assessment in Trauma, the authors were unable to determine a required number of exams to reach a good accuracy [18].

The principal limitations of this study were first, the recruitment of a convenience sample since this could not reflect the actual patients admitted to the ED for a suspicion of DVT. However, recruitment could only be performed during duty hours because of the local vascular laboratory availability and, when the ED were overcrowded, residents did not have time to recruit patients. Second, the number of exams per resident was low.

## Conclusion

In conclusion, despite reaching the main objective, this very short training session was inadequate to allow residents to exclude DVT with a sufficient security. A new training pathway with the addition of experiential phase will be deployed and evaluated.

## Data Availability

The raw data will be available upon reasonable request.

## Abbreviations

VLCU: Venous limited compression ultrasonography
DVT: Deep venous thrombosis
WLCU: Whole leg ultrasonography
NPV: Negative-predictive value
ED: Emergency Departments
EP: Emergency Physicians
POCUS: Point-of-care ultrasound
PPV: Positive-predictive value
